# Implementation of a text-messaging intervention for adolescents who self-harm (TeenTEXT): a feasibility study using normalisation process theory

**DOI:** 10.1186/s13034-016-0101-z

**Published:** 2016-06-28

**Authors:** Christabel Owens, Nigel Charles

**Affiliations:** University of Exeter Medical School, College House, St. Luke’s Campus, Heavitree Road, Exeter, EX1 2LU UK

**Keywords:** Self-harm, Text messaging, SMS, Adolescent, Child and Adolescent Mental Health Services (CAMHS), Normalisation process theory (NPT)

## Abstract

**Background:**

There are few interventions that directly address self-harming behaviour among adolescents. At the request of clinicians in Child and Adolescent Mental Health Services (CAMHS) in England and working with them, we redeveloped an adult SMS text-messaging intervention to meet the needs of adolescents under the care of CAMHS who self-harm.

**Methods:**

We used normalisation process theory (NPT) to assess the feasibility of delivering it through CAMHS. We planned to recruit 27 young people who self-harm and their clinicians, working as dyads and using the intervention (TeenTEXT) for 6 months.

**Results:**

Despite strong engagement in principle from CAMHS teams, in practice we were able to recruit only three clinician/client dyads. Of these, two dropped out because the clients were too unwell. We identified a number of barriers to implementation. These included: a context of CAMHS in crisis, with heavy workloads and high stress levels; organisational gatekeeping practices, which limited the extent to which clinicians could engage with the intervention; perceived burdensomeness and technophobia on the part of clinicians, and a belief by many clinicians that CAMHS may be the wrong delivery setting and that the intervention may have better fit with schools and universal youth services.

**Conclusions:**

User-centred design principles and the use of participatory methods in intervention development are no guarantee of implementability. Barriers to implementation cannot always be foreseen, and early clinical champions may overestimate the readiness of colleagues to embrace new ideas and technologies. NPT studies have an important role to play in identifying whether or not interventions are likely to receive widespread clinical support. This study of a text-messaging intervention to support adolescents who self-harm (TeenTEXT) showed that further work is needed to identify the right delivery setting, before testing the efficacy of the intervention.

## Background

Self-harm is defined as any “act of self-injury or self-poisoning carried out by an individual, irrespective of motivation” [1]. It takes many forms, the most common being cutting or burning of the skin and overdosing on over-the-counter analgesics. Self-harming behaviour tends to become habitual and, once established, patterns can be hard to break.

Self-harm is very common in children and adolescents, with prevalence peaking at 14–15 years [2]. UK school-based studies show that 13–14 % of 15–16 year olds report a lifetime history of self-harm [3, 4]. Studies consistently find higher prevalence rates in girls than in boys. When asked why they self-harm, adolescents most commonly report a desire to escape from intolerable thoughts and feelings, and wanting to punish themselves [5, 6]. Moran and colleagues comment that middle-to-late adolescence is characterised by problems of emotional control, and that biological changes taking place during puberty may undermine the ability to cope with stress and give rise to risk-taking behaviour [2].

Self-harming behaviour is associated with a ten-fold increase in risk of death by suicide [7], as well as with elevated psychopathology and increased demand for clinical services [8]. Effective management of self-harm may therefore save lives, as well as reducing the cost burden on healthcare systems [9, 10].

Most available interventions, including those showing the best early evidence of effectiveness, are designed to treat psychiatric co-morbidities, such as depression, rather than addressing self-harming behaviour per se [11, 12], and clinicians commonly complain that they have nothing in their toolbox to help clients with their self-harm.

In research with adults who self-harm, a range of contact-based interventions showed early promise. These involve either maintaining contact with individuals following a hospital episode through the periodic sending of supportive letters [13], postcards [14–16], telephone calls [17] or a combination of these media [18], or offering immediate re-entry to services in an emergency through the provision of a crisis card [19]. A recent systematic review and meta-analysis offers tentative confirmation that the sending of postcards may reduce the rate of repetition of self-harm in some adults [20]. Attempts to replicate this effect with adolescents have not been successful [21].

Text messaging offers a fast, convenient and low-cost alternative to letters and postcards and is likely to be more attractive to adolescents, especially those who are socially anxious, vulnerable and hard-to-engage [22]. Text-messaging systems have become widely used in the management of a wide range of long-term conditions and health-related behaviours [23, 24], including the delivery of health advice and support to adolescents with asthma, diabetes, coronary heart disease and other chronic conditions [25–27].

### Development of the intervention

In a previous study we worked with adults, using participatory methods, to develop a text-messaging intervention that would help them manage their self-harming behaviour [28]. Previous contact-based interventions for self-harm have involved the sending of generic messages at standard times [14, 15, 29], and are intended to be seen as a ‘gesture of caring’ by the service provider [30]. Our intervention differed radically from these insofar as it was designed as a self-management tool, which allows individuals to write their own messages and determine when to receive them [28]. Its unique features are personal content and personal timing. Drawing on elements of cognitive behavioural therapy (CBT), individuals are supported to write a set of self-efficacy messages or personal coping statements [31], which are stored electronically in a secure personal message bank and are delivered to the individual’s mobile phone at their own chosen times. Adult users reported that this helped them to feel in control, increasing self-esteem and reducing dependency on clinicians; three adults also reported that the timely arrival of a text-message had interrupted a suicide attempt and prompted them to reconsider whether they wished to die [32, 33]. In a meta-analysis of text-messaging interventions, Head et al. [24] demonstrated that those incorporating individually tailored messages and personal scheduling are more efficacious than those using standard content and scheduling.

We were subsequently asked by clinicians in local Child and Adolescent Mental Health Services (CAMHS) if we would adapt it for use by 12–18 year olds under the care of CAMHS. We consulted extensively with CAMHS teams at four sites and ran a series of creative workshops for adolescents who self-harm, inviting them to play with components of the intervention and help us tailor it to meet their needs. Researchers and software developers then worked closely with three clinicians from one CAMHS team to ensure that it was simple to deliver and fully addressed their concerns about risk.

The intervention requires users to write effective personal self-efficacy messages and to identify their own high-risk times. Prompted by clinical concerns, and because little is known about the capacity of younger populations to self-manage effectively [34–36], the adolescent version, known as TeenTEXT, was specifically designed to be used under the supervision of a CAMHS clinician.

### Aims and research approach

The aim of the study was to test and refine the intervention in situ, before proceeding to a full trial. Our research question was: Can TeenTEXT be administered by CAMHS clinicians within the context of everyday clinical practice?

Murray et al. urge researchers to consider at an early stage whether an intervention is capable of being ‘normalised’, i.e. widely implemented and integrated into routine practice [37]. They suggest that a preliminary study using normalisation process theory (NPT) can optimise intervention design, assess fitness for purpose and increase the potential for normalisation. If results suggest that the intervention has little prospect of implementation, it can then be abandoned before further time and funding are wasted on a full trial. NPT rests on four core concepts, which represent the conditions that are necessary for interventions to become embedded in everyday practice (Box 1). We used these to inform our study of the implementation process.

## Box 1: Core concepts in normalisation process theory (NPT)

### Coherence

This is about meaning and sense-making. Does the intervention make sense to practitioners? Do they understand its purpose? Is it clearly distinct from other interventions?

### Cognitive participation

This is about buy-in or commitment. Are practitioners willing to engage with the intervention and invest the time, energy and thinking required to change their practice?

### Collective action

This is the actual work of adopting the new tool or technology. What actions or behavioural changes are required and by whom? How do these affect, and how are they affected by roles, relationships, other areas of practice, resources and contexts?

### Reflexive monitoring

This is about appraising and making adjustments. Are practitioners convinced of the benefits of the new way of working? Do they find they need to modify the intervention in order to integrate it into everyday practice and make it sustainable?

## Methods

**Fig. 1:**
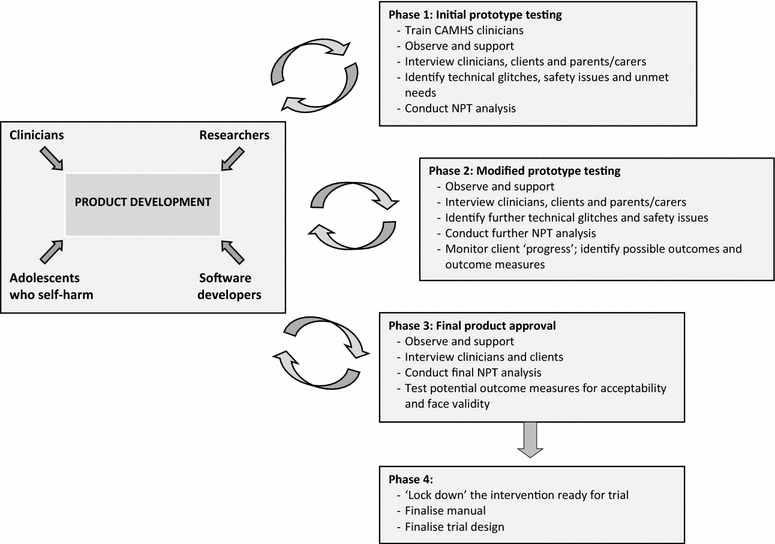
Study design: formative evaluation and feasibility of text-messaging intervention for adolescents who self-harm (TeenTEXT)

We developed a four-stage design, shown in Fig. 1, in which clinicians and their clients would work closely with the research team and software developers through a series of three iterations or feedback loops to optimise the intervention and assess whether it was sufficiently likely to normalise to be worth evaluating in a full trial. Ethical approval was given by the South West NHS Research Ethics Committee (REC 13/SW/0149).

### Settings and sample

We planned to test the intervention in three different CAMHS teams in South West England, recruiting three clinicians from each team (no of clinicians = 9), each of whom would identify three eligible clients from their caseload (no of clients = 27). This sample size was pragmatic. We wanted to work intensively with a small group of highly committed participants or product champions. Such individuals, who are willing to try out new innovations at an early stage and provide candid feedback, and who in return benefit from a high level of support from the product development team, play a key role in ensuring that new products are capable of being implemented in real-world contexts [38]. We envisaged that the three clinicians in each team would support and mentor each other for the duration of the study and subsequently cascade their knowledge down through the team, influencing others to adopt the intervention.

Adolescents were eligible to take part if they were CAMHS clients aged 12–18, had self-harmed on two or more occasions and recognised it as a problematic behaviour, owned a mobile phone and were able to write/read text messages in English. Parental consent was required for those under the age of 16.

### Delivering the intervention

TeenTEXT is made up of the following elements.

1. A workbook containing a series of exercises designed to help the young person develop their own personal messages and decide when to receive them. It includes examples of three different categories of message that emerged in the course of both the adult study and the development workshops with adolescents:

- ‘Things I can do to help myself’ (actions and distractions)
- ‘Accepting myself and how I’m feeling’ (validating emotions)
- ‘People who matter to me’ (reminders of social connectedness).

The workbook can either be completed in a CAMHS consultation or taken away and worked on at home, with the consent of the clinician.

2. A computer programme, hosted on a secure virtual server and accessed via a simple web interface on a PC, laptop, tablet or phone. Once the client and clinician have agreed on the content and timing of messages, the clinician logs into TeenTEXT, adds the client as a new user and is then able to enter the messages and set up a delivery schedule.

Two message delivery options are available: (1) specific messages can be scheduled to arrive at specific times that are known to be stressful or difficult, e.g. every Sunday at 6 pm; (2) if an unexpected situation arises and the young person needs a bit of support or encouragement, they can request a message by texting a given number and a randomly-selected message from their personal message bank is delivered immediately to their mobile phone. Three or more such requests in a 24-h period result in an alert being sent to their clinician. Content and timing of messages can be reviewed and adjusted by the client and clinician at each consultation.

3. A simple manual for clinicians to enable them to understand the basic functions of TeenTEXT and guide them through the process of delivery, with shorter versions for adolescents and parents/carers.

### Data collection and analysis

We wanted clinician-client dyads to use TeenTEXT for 6 months. During this time we planned to observe and support clinicians in setting up and monitoring client accounts and to make detailed field notes at each site visit, including thick description of the service contexts in which TeenTEXT was likely to be deployed. We also planned to conduct three rounds of individual semi-structured interviews with clinicians, clients and, where appropriate, parents/carers (see Fig. 1), in order to elicit their views on the possible benefits and risks of TeenTEXT and identify candidate outcomes to be measured in a subsequent trial.

Data collection was subsequently modified, as recruitment did not go as planned. We still collected field notes at the three sites, conducted a focus group with one full CAMHS team comprising 14 members, and conducted individual interviews with an additional seven clinicians and two service managers. The focus group and interviews were audio-recorded. All data were qualitative in nature and were subjected to inductive thematic analysis [39]. This involved the following steps: transcription; familiarisation; coding and sorting of units of data into meaningful categories based on a set of preliminary themes, and finally the generating of broader interpretive themes informed by NPT, which were used to structure this report.

## Results

**Fig. 2:**
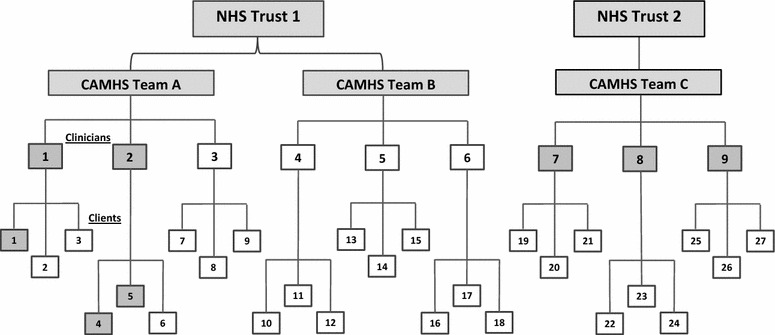
Planned and actual recruitment. *Shaded boxes* represent those recruited, out of planned totals

After 12 months of strenuous engagement activity in three CAMHS teams and two NHS Trusts, only three clinician-client dyads had been recruited. Of these, two dropped out quickly because the clients turned out to be too unwell. One client used TeenTEXT with the support of a clinician for 4 months, before being discharged from the service, aged 18, and moving away. Figure 2 depicts the proposed sample, with shaded boxes representing the numbers that were actually recruited. In CAMHS Team C, three clinicians were recruited late in the study and were very keen, but further delays caused by sickness and annual leave meant that there was insufficient time for them to use it with their clients.

Instead of collecting data as originally planned, we therefore focused our attention on trying to understand the barriers to recruitment and implementation. The NPT-informed themes that emerged from the data are presented below.

### Engagement in principle

Wherever we presented TeenTEXT, clinicians and managers alike were agreed that it made sense and was immediately appealing. Clinicians quickly grasped the basic principles and saw it as a potentially valuable tool to help young people manage their self-harming behaviour and the persistent negative thinking and negative self-evaluation that go with it. They saw it as complementing existing approaches, such as Cognitive Behavioural Therapy (CBT) and Dialectical Behavioural Therapy (DBT), and could see how TeenTEXT could reinforce the learning from them:

*“I like the fact that the messages are written by them, so they’re supporting themselves… This fits with what we currently do, which is try and give them a sense of control.” (ID:04)*

Some clinicians saw it as being particularly useful to sub-groups with specific communication difficulties, such as deaf young people or those with autistic disorders. Others recognised its potential use in the management of behavioural problems other than self-harm, such as eating disorders.

In NPT terms, the coherence of the intervention was never questioned. This was unsurprising, given that it had been developed at the request of, and in partnership with, CAMHS clinicians. However, it made it all the more surprising that, in practice, so few were willing to try it out with their clients.

### Context: CAMHS in crisis

At the time of recruiting, two CAMHS teams that had been involved in early consultations were undergoing wholesale reorganisation and were therefore unable to participate in the feasibility study. Another CAMHS service had recently been privatised and was without any research governance structures.

Three clinicians from Team A (Fig. 2) had worked with us in the development of TeenTEXT and had all been keen to try it out with their clients. However, by the time we came to recruit to the feasibility study, one was on long-term sick leave, one on maternity leave and one was no longer in post. All the CAMHS teams were experiencing very high levels of staff sickness, work-related stress and burnout. Interview participants reported caseloads that were twice the size they should have been and a system under enormous strain:

*“CAMHS is overwhelmed at the moment… It may have been the wrong time to try something new… There have been so many organisational changes. Managers have left, there’s been the introduction of Child IAPT*^1^*services and there are high rates of sickness absence. This does affect our ability to get involved with new projects.” (ID:02)*

*“We’ve had two new line managers in the last six months, and they need to be on board for anything new to happen.” (ID:07)*

### Organisational gatekeeping

Possibly the most significant barrier to implementation, particularly within a research context, was the need for buy-in at management levels and the time it took to obtain this. Individual clinicians had participated in the development of TeenTEXT at their own discretion. When it came to implementing the intervention, however, management approval was essential. Despite having full NHS research ethics approval, research governance approval and unequivocal support from the Heads of Children’s Services in both NHS Trusts, operational managers were wary. Months went by while we waited for meetings to be arranged, attended meetings and allayed fears, seemingly going over the same ground again and again. One informant confirmed this:

*“The organisation doesn’t give clinicians any leeway. We need permission to try anything new and there are so many hoops to jump through before that happens.” (ID:05)*

Even then, it was difficult to gain access to clinicians. In each team, we had hoped to invite clinicians to a hands-on session, with a demonstration of TeenTEXT, an opportunity for them to play with it and plenty of time for questions. The pressures under which teams were working meant that this was simply not possible. In one team, we were given a 20-min slot in which to introduce the project to clinicians. It was just one item on the agenda of a general team meeting, which offered no opportunity for a practical demonstration, and there was no possibility of arranging a follow-up session. In another team, managers insisted on circulating information to clinicians via e-mail and managing the recruitment process on our behalf. Not one clinician was recruited from that team (Fig. 2).

In NPT terms, this severely limited the level of cognitive participation we were able to achieve. Clinicians were not given sufficient opportunity to engage with the intervention and think about whether and how they could incorporate it into their practice. As they commented:

*“TeenTEXT never really got onto our radar.” (ID:09)*

*“It needed the managers to be on board and for them to give us [clinicians] the time to think about it and discuss it internally and with the researchers.” (ID:03)*

### Perceived burdensomeness and technophobia

In the context of very heavy caseloads, high stress levels and exhaustion, the effort involved in mastering a new technology and incorporating it into everyday practice was perceived to be too much by clinicians. Although some reported that they were using apps of various kinds with their clients, others appeared to be resistant to technological interventions:

*“The general perception within the team is that using TeenTEXT is too much of an extra burden on top of our existing workload.” (ID:03)*

*“It feels like there’s a lot to learn, especially for non*-*IT literate people.” (ID:04)*

These views were not based on any knowledge or experience of using TeenTEXT but on a preconception, which might have been corrected if we had been able to organise a practical session and allow them to try it for themselves. The clinician who used it with two clients found it simple to use:

*“It hasn’t been difficult or too time*-*consuming. I have had to allocate time, but I’ve chosen to prioritise it because I could see that it would be good for the young people I work with.” (ID:09)*

### Right intervention; wrong setting

Despite the fact that the impetus for the development of the intervention came from CAMHS clinicians and that it had been developed with them, nearly all informants believed that CAMHS was not the ideal delivery setting.

All commented on the high threshold for CAMHS, which means that they see only the most acute and complex cases. Whilst many of their clients self-harm, it is often overshadowed by other problems, including anxiety and depression, emergent personality disorder, excessive alcohol or illicit drug use and risky sexual behaviour, and may not be a treatment priority. Clients are often so unwell that clinicians struggle to engage with them at all.

Furthermore, duration of contact with CAMHS is typically short. Services are under pressure to discharge clients as quickly as possible, due both to long waiting lists and to a clinical desire to avoid dependency. Several clinicians reported that they would not usually have enough sessions with a young person to enable them to set up and use TeenTEXT, and they identified a need for robust arrangements to be in place for handing over the monitoring of a client’s TeenTEXT account to another agency or non-specialist service following discharge. This view was supported by the one CAMHS client who did use the intervention very successfully for 4 months and regretted the fact that it had to be withdrawn when s/he was discharged from CAMHS.

Clinicians all pointed out that only a very small percentage of young people who self-harm are seen by CAMHS. For all these reasons, informants believed that the intervention might be better delivered in other settings, such as schools and youth services, where it could be used to help young people gain control of their self-harm at an earlier stage and prevent it from escalating:

*“It doesn’t fit our short*-*term model of working with young people where interventions may only be for two months.” (ID:01)*

*“We see young people with severe mental health problems, including suicidal ideation, and I’m not sure it’s ideal for this group… Most self*-*harm is dealt with by family support workers and schools, and they are always looking for additional resources and tools to help with it.” (ID:08)*

## Discussion

We achieved strong engagement in principle from CAMHS teams, but limited engagement in practice. Clinicians all understood the purpose of the intervention and recognised that it could be valuable in the management of self-harm and other problem behaviours, but heavy workloads, high stress levels and possibly some technophobia contributed to a perception that too much effort was required to master it and incorporate it into their practice. Time pressures and organisational gatekeeping made it difficult for us to persuade them otherwise through hands-on demonstration sessions. There was also a strong belief that most CAMHS clients were so acutely unwell that they would struggle to engage with it. This was confirmed by the fact that, of the three young people who opted to use TeenTEXT, two turned out to be too unwell to do so.

The clinicians who were involved in early consultation and intervention development had not identified any of these issues. User-centred design principles and the use of participatory methods in intervention development are therefore no guarantee of implementability. Barriers to implementation cannot always be foreseen, and early clinical champions may overestimate the readiness of colleagues to embrace new ideas and technologies. This may be particularly true in areas of clinical practice such as self-harm, where there are few effective interventions and there is a strong desire among some clinicians to find novel solutions.

Interpreting our findings using NPT terminology, there was good coherence, limited cognitive participation but no collective action, and therefore no opportunity for reflexive monitoring by intervention users.

The context in which clinicians were working certainly did not help. Cognitive participation is a key stage in implementation. No matter how promising an intervention looks from the outside, it will not work unless there are enough individual actors who are willing, and feel able and supported, to invest the time and effort required to master new techniques and incorporate them into their practice. This may be particularly difficult to achieve in times of rapid change, service re-organisation and workload crisis. In the context we have described, it is unlikely that any new intervention would have gained widespread support. Clinicians were struggling to deliver the known and familiar, and simply did not have the capacity to embrace the novel.

Research and innovation are enshrined in the constitution of the NHS in England [40], but the structures in which individuals operate on a day-to-day basis, and the requirement for practice to be evidence-based, may stifle their freedom to experiment with new ideas and technologies. Our study demonstrates the importance of obtaining buy-in from operational managers, but managers were looking for evidence of effectiveness before sanctioning new practices: a Catch-22 situation. Early formative research and feasibility studies may be perceived as involving more risk to organisations than later randomised controlled trials.

The academic context was also challenging. Long delays between intervention development work and the feasibility study, incurred whilst applying for funding and awaiting decisions, resulted in the loss to the project of whole clinical teams and several key clinical champions (see Box 2). Short-term funding made it difficult to build and maintain the secure, long-term relationships with clinical teams that are essential in this kind of work.

Previous studies of contact-based interventions for self-harm involving the delivery of supportive letters, postcards, phone calls and text messages [15, 17, 18, 21, 29] have not only used generic messages and standard scheduling, but have also used researchers to do the work of delivering the intervention, i.e. sending the postcards. Whilst this may demonstrate an effect, it does not show that the intervention is sustainable once the study has ended. Little is known about the capacity of staff in clinical services to take on these additional tasks once the research is over. An important strength of our study was our commitment to testing the intervention ‘for real’, with the work of intervention delivery being performed by those who would ultimately be responsible for it.

It is possible that the region in which we tested TeenTEXT is atypical and that other CAMHS teams might have embraced it more readily. The geography of the South West of England poses particular challenges for CAMHS, inasmuch as small teams provide services to very large rural areas and clinicians spend a large amount of time driving. The fact that three members of Team C were very keen but were recruited too late to participate in the study indicates that the intervention may still have a place within CAMHS, but further work is clearly needed to identify the right delivery setting, before testing the efficacy of the intervention.

## Box 2: Key learning points for researchers

- Researchers should not underestimate the strain under which clinical services may be operating and early discussions should focus specifically on the capacity of staff to participate.
- Obtaining buy-in from operational managers and senior staff is essential and plenty of time should be allowed for this.
- Beware of organisational gatekeeping and insist on giving a practical hands-on demonstration of the intervention to clinicians.
- A clinical champion within each team is also a critical success factor. Ideally, this should be someone who has been closely involved in intervention development, who can enthuse colleagues and reassure them about workload demands. If, as in our case, your clinical champions are on long-term leave, consider postponing the study until they are available or can be replaced.

## Conclusions

This study demonstrates the challenges of implementing a text-messaging intervention to support adolescents who self-harm (TeenTEXT) within CAMHS. It confirms that NPT studies have an important early role to play in identifying problems in proposed delivery settings that may affect the likelihood of an intervention receiving widespread clinical support and being integrated into routine practice. Our study contains an important lesson for those developing and trialing interventions of all kinds, namely that they ignore implementation contexts at their peril.

## Footnotes

1 Improving access to psychological therapies.
